# Men perspectives on attending antenatal care visits with their pregnant partners in Misungwi district, rural Tanzania: a qualitative study

**DOI:** 10.1186/s12884-021-03585-z

**Published:** 2021-01-28

**Authors:** Maendeleo Boniphace, Dismas Matovelo, Rose Laisser, Hadija Swai, Victoria Yohani, Sylvia Tinka, Lusako Mwaikasu, Hannah Mercader, Jennifer L. Brenner, Jennifer Mitchell

**Affiliations:** 1 Catholic University of Health and Allied Sciences (CUHAS), Mwanza, Tanzania; 2 Bugando Medical Centre, Mwanza, Tanzania; 3 University of Calgary, Calgary, Canada

**Keywords:** Male attendance, Pregnant partners, Antenatal care, Rural-Tanzania

## Abstract

**Background:**

Mens’attendance with their pregnant partners at facility-based antenatal care (ANC) visits is important for maternal and child health and gender equality yet remains uncommon in parts of rural Tanzania. This study examined men’s perspectives on attending ANC with their pregnant partners in Misungwi District, Tanzania.

**Methods:**

Twelve individual interviews and five focus group discussions were conducted using semi-structured questionnaires with fathers, expectant fathers, and in-depth interviews were done to health providers, volunteer community health workers, and village leaders. Interviews were recorded and transcribed in Swahili and later translated to English. The research team conducted thematic analysis to identify common themes among interviews.

**Results:**

We identified two broad themes on the barriers to male attendance at facility-based ANC visits: (1) Perceived exclusion during ANC visits among men (2) Traditional gender norms resulting to low attendance among men.

**Conclusion:**

Attendance at health facility for ANC visits by men with their pregnant partners in the study areas were challenged by structural and local cultural norms. At the facility men were uncomfortable to sit with women due to lack of specific waiting area for men and that they perceived to be neglected. Local cultural norms demanded women to have secrecy in pregnancy while men perceived not to have a role of being with their partners during ANC visits.

## Background

Maternal mortality remains a problem worldwide, especially in Africa. Globally, more than 800 women die every day from preventable causes and women from resource poor settings are most at risk [1]. In Sub-Saharan Africa, 1 in 38 women die due to preventable or treatable complications during pregnancy and delivery [2]. Care during pregnancy (antenatal) and delivery by skilled health providers at a health facility are associated with reduced maternal and child morbidity and mortality [3, 4].

Gender equality is recognized as an important social determinant of health. Involvement of men in maternal and child health is core to gender equality especially its impact on maternal and child health outcomes. Male responsibility in transforming social norms towards health and child development, including taking responsibility for reproductive health issues is critical [5]. This call to action has been emphasized since the International Conference on Population and Development [6] and the Fourth World Conference on Women [7] and remains a priority today towards gender equality targets for the 2030 Sustainable Development Goal Agenda. Increased male engagement in maternal child health can increase shared decision making around impactful health choices such as parenting, health care-seeking for delivery and illness, contraception and family planning. Increased male involvement during pregnancy reduces maternal stress via emotional, logistical, and financial support [8, 9]. Male participation at ANC visits is associated with increased use of delivery and postnatal health services and reduced postpartum depression [10].

In African culture, males are often the key family decision makers, including decisions related to whether, when and where a pregnant woman should begin ANC services. In Nigeria and Ethiopia, women who made the decision to attend ANC jointly with their husbands/partners were significantly more likely to attend the recommended four or more antenatal visits compared with women whose husbands/partners made family decisions alone [11–13]. Similarly, in Eritrea and Ethiopia, women involved in family decision making, were more likely to attend more and earlier ANC services at health facilities [14]; women without shared decision-making often did not then not attend ANC visits until their third trimester [15].

Cultural norms and social economics influence a pregnant woman’s access to health services and where delivery occurs. Traditional beliefs related to pregnancy and childbirth in communities may be longstanding such as ‘a long labor indicates a marital affair.’ Thus, women may not want others to observe the length of their labor and pass judgment and this may impact a decision to deliver at home [16]. One southern Tanzanian study reported home deliveries were due to women’s reluctance to make their own decision and compounded by a lack of money [16]. Often, the decision about delivery location was made by the pregnant woman’s mother, mother-in-law or husband [16].

Many African countries and communities have made efforts to increase male engagement in maternal and newborn health. In Malawi, one program involves peers who encourage each other [17, 18] and in Uganda, education programs share the positive experiences of women who delivered by Skilled Birth Attendants [19]. In Ghana, reproductive health campaigns emphasized the importance of responsible sexual behavior, small family size and mutual respect for women. The education program in Ghana has led to reduced total fertility and mortality rates and contraceptive prevalence rates have been increasing steadily over the years [20].

While some studies have explored the barriers around male involvement in African countries, we sought to build on this literature by looking specifically at perspectives of men on their participation in ANC visits. We focused on local rural communities in Misungwi district, Lake Zone, Tanzania where maternal and child mortality is amongst the highest in the country and where participation of men in ANC is limited. This study builds on a qualitative 2016 survey in which women respondents identified that the lack of male engagement was a barrier to attending at the health facility-based ANC.

## Methods

This qualitative study intended to deepen understanding of male perspectives about their attendance at facility-based ANC visits together with their partners. This study was nested as a sub-study within a larger longitudinal implementation and evaluation of the Mama na Mtoto project intervention in rural Tanzania, which aimed to improve the delivery of essential health services to pregnant women, mothers, newborns, and children under five in Misungwi and Kwimba Districts.

### Study setting

Misungwi District is among seven districts of Mwanza region. It consists of 2579 km^2^ with population of, population 351,607according to the national census 2012 (NBS,2012). The district is located in the Northwestern part of Tanzania, 45 km from Mwanza town. The district has a predominantly rural population (91%) and a majority population are Sukuma, (91.9%,) speaking their tribal language in addition to Swahili (Tanzania National language). The District is divided into four divisions two urban and two rural. The major economic activities are cattle-keeping and subsistence farming. Misungwi district has 2 hospitals, 4 health centers and 45 dispensaries. Two communities (‘divisions’) were selected for study, due to their high maternal mortality [21].

### Study design

This qualitative study was informed by the Ecological framework [22] and the framework influenced sampling, data collection and analysis. Ecological frameworks consider the individual, interpersonal, community and societal factors and recognize the complex interplay across all levels of a health problem and the influence on health behaviors. Using an ecological framework sensitized our examination of barriers to male attendance to ANC to the multiple factors impacting attendance.

### Sampling procedure

Interview and focus group participants were recruited using purposive sampling method whereby from the four divisions of Misungwi district we purposively choose two rural divisions (Mbarika and Innonelwa) based on its unfavorable MNCH indicators [21]. In the divisions we conveniently selected one ward in Mbarika and three wards in Innonelwa. At the ward level sampling procedures were culturally sensitive and tried to foster safety and trust in the communities. As such the following steps were taken:

- First, five villages (one in Mbarika and four in Innonelwa) were chosen based on geographical convenience.
- Initial contact was made with the village executive officer (local leader) in the village/ community to explain the purpose of the study.
- The local leader arranged orientation meetings with village officials and community health workers (CHW).
- Next a public meeting was held to inform community members about the study aim and selection criteria of participants. The meeting was intended to build trust, outline participant criteria, and ensure voluntary participation.
- Criteria for participation included being a male over the age of 18, who had a partner experiencing their first pregnancy, males with one or more children, no cognitive disability, and a permanent resident living in Misungwi district for over 6 months. Key informants selected included village leaders (potential influencers of health service uptake), volunteer CHWs, and health providers working at local primary care facilities.
- Meetings were held with participants who had volunteered to participate. Consent was discussed and documented, including confidentiality of the data and the right to withdraw at any time. Dates and locations of interviews were discussed with participants.

### Data collection

The research team, developed an interview guide in advance incorporating personal experience of team members, relevant literature, and questions aimed to target different levels of the ecological model. This tool was piloted in a different but similar rural environment with men of similar characteristics, and small modifications and probes were added to the guide. Questions included “how does your community perceive men who attend ANC appointments with their partners?” and “what were your experiences or what have you heard about attending an ANC visits?” Sukuma-speaking research assistants were recruited to assist in obtaining consent and data collection for non-Swahili speaking participants. In total, five focus group discussions and 12 in-depth interviewers were conducted with a total of 50 participants.

#### Focus groups discussions (FGD)

The five FDG were composed only of fathers and homogenous by age to promote comfort and build on common emergent themes [23]. The focus groups lasted on average of 60–90 min and took place at quiet and convenient places agreed by participants in their locations/homes. Each FGD consisted of 8–12 participants. There were 15 men whose partners were pregnant for the first-time and 29 fathers who had one or more children. Their ages ranged from 25 to 60 years old.

#### Individual interviews

Individual interviews were conducted with one health care provider; three village leaders and two Community Health Care Workers (CHWs). The individual interviews lasted on average of 40–60 min each and took place at participants’ homes or at a secured room in the village office as per participants’ choices. Additionally, fathers from the FGD were selected for individual interviews to provide more in-depth feedback to meet saturation. Six men took part making a total of 12 Individual interviews conducted.

In both FGD and IDI, the facilitators conducted semi-structured interviews with participants, interviews were recorded, and research assistants wrote field notes and documented non-verbal cues that provided a secondary source of data. Interviews conducted in Swahili were transcribed and then translated into English while those in Sukuma were transcribed in Kiswahili translated into English. All approached participants who agreed to join the study participated.

### Quality checks

Quality checks for the transcripts were performed by research team members who were not involved in data collection through listening to audio and reading corresponding transcript and noting any errors. Data were later reviewed by researchers who had conducted the interviews; they re-read all transcripts while listening to the audio recordings for the purpose of further validating the transcripts for accuracy and language.

### Data analysis

Data analysis began during interviews and team meetings after the interviews. Team members reviewed data regularly to identify emergent themes and add questions or probes to build on and deepen preliminary themes. Saturation occurred when repetition was noted, and no additional themes were found. The research team used thematic analysis [24] to identify themes among all interviews. Analysis began with familiarization of the data through reading and listening to audio. Next, as research analysts read all transcripts line by line, the data were labeled with a word or phrase (code) that described the data and the code was entered into the data management program, Nvivo V.12. Open coding was initially conducted, with later readings and coding informed by the ecological model [22] and relevant literature. For example, reading specifically for data on interpersonal factors such as family dynamics or community factors such as bylaws related to maternal care. Codes were collated and then sorted into broader themes. Memos were made to describe the rationale and process of sorting codes into the themes. Research analysis discussed discrepancies about themes until consensus was achieved. The main themes were obtained upon agreement with the research team.

### Member checking

Participants were contacted 6 months later and invited to attend a meeting in neutral venue in Misungwi to review the preliminary themes. Of the 50 participants, approximately 18–20 attended the meeting. Preliminary results were presented to the group and participants were asked to confirm key themes and provide any missing information which was not captured in the themes. Participants shared their agreement on the presented themes. No missing or exaggerated as per member checks.

## Results

The table below shows demographic characteristics of participants (Table 1).

**Table 1:** Demographics of study participants (*N* = 50)

Characteristics	N	%
Age		
25–34 years	15	30
≥ 35 years	35	70
Highest education level achieved		
Primary	41	82
Secondary (I-IV)	8	16
High (Form VI+)	1	2
Occupation/category		
Peasant	42	84
Village leader	2	4
CHW	2	4
Health provider	2	4
Pastor	1	2
Other (driver)	1	2
Marital status		
Married	40	80
Common law relationship	10	20

Two broad themes were identified in the data: (1) Perceived exclusion during ANC visits among men (2) Traditional gender norms resulting to low attendance among men.

Men reported inconsistent experiences when attending ANC visits together with partners. While Tanzanian government directives recommend that pregnant women who attend ANC services with a male partner will be given priority, this practice was inconsistent.

There are no priorities [silence] … when you are at the clinic, provider’s help who comes first and after you are done with the examination with the provider, you will be given the card for you to come back next visit. That means there is no priority. (Expectant father)

Some men reported they were or feared they might be excluded from interactions with health providers during the ANC appointment. Men described choosing not to escort their partners since most facilities lack the physical space to accommodate an additional attendee during assessment. Often they described being left outside a facility during ANC while their wives/partners received service within the building. One father shared, “*The challenge is when you go to the clinic with your wife, they enter her in the room, and then you stay outside like a watchman of the bicycle [laugh]” (father with more than one child).* Men shared that they felt their time was wasted when they could not attend in the examination room:

“But there is one challenge that, when you escort your wife for ANC, when you reach the health facility you can find … no chairs or space … you just remain walking around the clinic and no one is considering you. Your wife is busy with a child and clinic services but for me, you are just wasting your time for walking around the health facility and exchanging ideas with your fellow men, this makes us not want to accompany our wives to the clinic” (expectant father)*.*

Beyond the physical space, men who attended reported receiving limited or no information regarding their partner, pregnancy progress or other pertinent medical information during the visit. Many men shared that they had expected to be actively included in the checkup:

… I want to be involved in the examination room and to be given findings [laugh] but not for me to remain outside … when I reached the clinic, they call my wife’s name, then they leave me outside … men have to go with their wife in examination room in order to hear the results she is told, if it is nutrition and if it is a lack of blood I need to know and not wait only her to tell me. What if she forgets other important things … so in my opinion men need to be in examination room (father with more than one child)

### Traditional gender norms resulting to low attendance among men

This theme revolved around common or local cultural beliefs and traditional gender roles in the surrounding community. Included were two subthemes: (a) Secrecy, and (b) Shame. It was reported that in the Sukuma community, men most often work outside the home with women fulfilling the household labour. Pregnancy, childbirth and childcare are often categorized as “women’s work.” Many men reported that as a result of their work-related roles their time is better spent working, rather than attending ANC appointments:

What causes men not to escort their partners first is economy. Our economy is poor, and you cannot go together at [ANC] clinic if you do not have something for consumption at home … when we go together, what will we eat after coming back from the clinic? (Father with one child)*.*

- **Secrecy:** Men described that it was common practice for a woman to keep her pregnancy a secret from her partner. Even when pregnancy is disclosed to a partner, women may keep pregnancy progress details a secret, reducing a male partner’s ability to engage fully in maternity issues and care. One expectant father shared his experience:
  … they do not tell us, their husbands, that is what am seeing. When she comes from the [ANC] clinic she will not even communicate to her husband. She might be told to tell her husband [cough] but she will not … we are seeing that in our households--they don’t give us information (expectant father)*.*

Fathers whose partner/wife were pregnant and community in-depth informants recounted this practice. According to one health provider*, “you may find women are secretive, they don’t put things open to their men, they don’t give them information” (health provider).*

(b) **Shame:** Men report feeling shame, stigma and resistance related to ANC attendance. Many men perceive that the act of attending ANC will be interpreted by others as being dominated or “whipped” by their wife/partner. One father explained:
  We escort our wives to clinic but everyone has his or her perception; others will perceive that you are right, others will judge you negatively by saying … "This man has been charmed by his wife”. (Expectant father)

Participants described that in the (majority) Sukuma tribe, a male walking together with his female partner is uncommon. One village leader explained: *For us Sukuma people we are not used to walk long distance with partner; you let her go first and then you come after she has reached a certain distance. We feel ashamed; traditionally that is what we are used to.*

One father explained how ‘educated’ versus ‘traditional’ views might be differently associated with perceptions around male engagement in ANC:

There are two categories in this community; those who are somehow educated and those who are too traditional. For the educated category, the act of escorting your partner to ANC services is regarded as caring and loving and is seen as normal but for the other group of men escorting their partner to the clinic is regarded as someone who has been whipped by his wife (father with one child)

Furthermore, shame and discomfort were reported by men owing to being surrounded by women while waiting at the facility. For example, men reported embarrassment and feeling as though they did not belong:

You find at the clinic you’re only one or two men alone while seated with many pregnant women around you, you feel ashamed … you get fear of coming next time because you’re alone in the bench sitting with many women ( -Father with more than one child)

Others reported men may feel ashamed attending ANC with partners if they perceived themselves and their partners to not possess good clothing or not having the financial ability to provide the recommended delivery supplies. Men described fear of others’ judgement and being mocked:

*There are some men in this community who don’t want to escort their partners to clinic because their partners do not have proper clothes. Men fail to buy nice clothes for their partners and when the time of going to clinic arrives you find that the woman has no proper attire thus making the husband ashamed of going with his wife (Father with more than one child).*

## Discussion

The findings of this study reflect male perspectives and candid experiences from a rural Tanzanian district on adherence to the importance of male participation in facility ANC services with their partners. These results indicate inconsistent experiences among men’s attendances to ANC where men reported being left out during an ANC service. For example, on reaching the clinic men reported to have no place to sit, not allowed to be with their partner in examination room during ANC examination. A social cultural norm that pregnancy matters are for women made men to report being uncomfortable in a group of women at the clinic. A feeling of shame reported to occur among men, most of them due to low economic status resulting to poor clothing and having been seated with many women while men are few at the health facility. A norm that pregnancy is a hidden affair made women not to talk about it with their husbands, resulting into more secrecy on pregnancy details to their husbands. Also, men explained that escorting your wife/partner to ANC is considered as love and caring especially for the educated men. The findings from this study suggests encouraging male to attend to ANC with their partners in rural setting in Tanzania. Although, males attending with their pregnant partners is increasingly being promoted as an expected standard and through to promote optimal family-centered care, its achievement in many communities, including much of rural Sub-Saharan Africa, has been hard to achieve [9].

Our study presents some ANC male engagement barriers which may be unique to the setting (i.e. Sukuma ethnicity), our key themes are consistent with findings from elsewhere [9, 25] However, other women reported preference for attending health services to include an environment which allow them to speak freely with their fellow pregnant women without a male partner [18]. Consideration of comfort for both males and females in the context of local culture, might promote innovations at facilities to better accommodate joint engagement.

Our study is also related with other studies from Sub Sharan Africa which have identified unique cultural and gender norms serving as barriers to male attendance to ANC service affecting male engagement advantages [26–29]. These affect male engagement beyond the pregnancy period [30, 31]. Rural Ghanese participants [31], similarly, reported the importance of men continuing to work rather than spending full days at a clinic; Furthermore similar logistical reasons of poverty, scarcity of work and traveling for work were raised. Such economic difficulties intersect with and can compound gender roles often furthering the division of spheres of home and work. Yet, while it is men who are making the decisions or who may be providing transportation or finances, it is evident that pregnancy is not solely a woman’s issue. Hence, there is need to work on male engagement challenges in the study area.

The strength of our study is that it has considered rural population where ANC seeking for men is not favorable [21] compared to other studies which were done in the urban. Qualitative methods with IDI and FGD data collection approaches allowed us to explore men’s perspectives at their own setting on a topic which is culturally not considered to be their role. Despite that we gained men’s perspectives from few men and few health workers we reached saturation of information.

## Conclusion

Male attendance at ANC with their pregnant partners in this study is affected by local beliefs, that male escorting their partners is being dominated (whipped) by their wives/partners. Health care workers and pregnant women need to communicate pregnancy progress to men escorting their partners after ANC examination. However, promoting male engagement during ANC is very critical to enhance ANC attendance for both partners in rural settings in Tanzania.

## Recommendations

This study suggests communities to encourage male involvement incorporating locally relevant strategies, such as instituting of local bylaws as catalyst for fostering attendance. Local government meetings should in-cooperate the agenda to discourage unfavorable gender norms, which demotivate male involvement. Community health care workers and male champions (educated men) to educate men on the importance of attending ANC with their partners. The health facility management to need to consider clinic environment which supports men’s needs during ANC.

## Data Availability

The datasets used and/or analyzed during the current study are available from the corresponding author on reasonable request.

## Abbreviations

CUHAS: Catholic University of Health and Allied Sciences
MNCH: Maternal and Newborn Child Health
ANC: Antenatal Care
PNC: Post-natal care
IDI: In-depth Interviews
FGD: Focus Group Discussion
MnM: Mama na Mtoto
VEO: Village Executive Officer
CHW: Community Health Workers
